# Dysregulation of autophagy in the central nervous system of sheep naturally infected with classical scrapie

**DOI:** 10.1038/s41598-019-38500-2

**Published:** 2019-02-13

**Authors:** Óscar López-Pérez, Alicia Otero, Hicham Filali, David Sanz-Rubio, Janne M. Toivonen, Pilar Zaragoza, Juan J. Badiola, Rosa Bolea, Inmaculada Martín-Burriel

**Affiliations:** 1 Laboratorio de Genética Bioquímica (LAGENBIO), Universidad de Zaragoza, IA2, IIS Aragón, Zaragoza, 50013 Spain; 2 Centro de Investigación en Encefalopatías y Enfermedades Transmisibles Emergentes, Universidad de Zaragoza, IA2, IIS Aragón, Zaragoza, 50013 Spain

## Abstract

Autophagy is a dynamic cellular mechanism involved in protein and organelle turnover through lysosomal degradation. Autophagy regulation modulates the pathologies associated with many neurodegenerative diseases. Using sheep naturally infected with scrapie as a natural animal model of prion diseases, we investigated the regulation of autophagy in the central nervous system (CNS) during the clinical phase of the disease. We present a gene expression and protein distribution analysis of different autophagy-related markers and investigate their relationship with prion-associated lesions in several areas of the CNS. Gene expression of autophagy markers *ATG5* and *ATG9* was downregulated in some areas of scrapie brains. In contrast, ATG5 protein accumulates in medulla oblongata and positively correlates with prion deposition and scrapie-related lesions. The accumulation of this protein and p62, a marker of autophagy impairment, suggests that autophagy is decreased in the late phases of the disease. However, the increment of LC3 proteins and the mild expression of p62 in basal ganglia and cerebellum, primarily in Purkinje cells, suggests that autophagy machinery is still intact in less affected areas. We hypothesize that specific cell populations of the CNS may display neuroprotective mechanisms against prion-induced toxicity through the induction of PrP^Sc^ clearance by autophagy.

## Introduction

Transmissible spongiform encephalopathies (TSEs), or prion diseases, are a group of fatal neurodegenerative disorders that can affect humans and animals^1^. TSEs include kuru, Creutzfeldt-Jakob disease (CJD) and its variant, Gerstmann-Sträussler-Scheinker (GSS) disease, and fatal familial insomnia in humans, bovine spongiform encephalopathy in cattle, and scrapie in sheep and goats^2^. Scrapie was the first TSE known and can be considered a good natural animal model to study the neuropathological mechanisms of these diseases^3^.

Prion diseases are characterized by a rapidly progressing course that leads inevitably to death, usually within a few months. According to the protein-only hypothesis^4^, TSEs are caused by the conversion of the normal mammalian cellular prion protein (PrP^c^) into its pathological conformation, or scrapie-associated prion protein (PrP^Sc^), which is abnormally folded, β-sheet enriched and partially protease resistant. Hence, prion diseases share profound similarities with other protein misfolding and neurodegenerative diseases like Alzheimer’s, Huntington’s and Parkinson’s disease^5^. The accumulation of PrP^Sc^ in the central nervous system (CNS) induces neuronal degeneration, vacuolation of the neuronal cell bodies (intraneuronal vacuolation) and neuropil (spongiosis), glial cell activation and neuronal loss by cellular death^6^.

Although several mechanisms have been proposed to explain neuronal death in prion diseases, apoptosis and autophagy are the types of cell death considered most likely to be involved^7^. Studies on the molecular mechanisms underlying neuronal apoptosis in brains of ovine naturally infected with scrapie have shown that, besides the upregulation of the pro-apoptotic protein BAX (BCL2 Associated X, Apoptosis Regulator) and its correlation with neuropathological features of scrapie, this process appears to be blocked somehow, or it is present at extremely low levels^8,9^. Apoptosis arrest could be a consequence of the activation of neuroprotective pathways that counteract massive cell death.

Autophagy is a fundamental cellular process involved in the turnover of long-lived proteins, protein complexes, cytoplasmic constituents and whole organelles through lysosomal degradation, in response to external and internal triggers. One of the primary roles of autophagy is to respond to nutrient starvation by producing amino acids^10^. Besides this fundamental role, autophagy contributes to other physiological processes such as intracellular clearance, differentiation, organismal development and elimination of invading pathogens^10,11^. Paradoxically, despite these pro-survival functions, autophagy can also mediate a non-apoptotic cell death, also called autophagic cell death^12^. When properly regulated, autophagy supports normal cellular and developmental processes, whereas autophagic dysfunction is associated with several pathologies, including neurodegenerative disorders^13^. It is still difficult to decipher whether active autophagy in the degenerating neurons plays a protective role, contributes to pathogenic neuronal death, or both.

Autophagy seems to be the main route of PrP^Sc^ degradation^14^ and autophagic vacuoles have been described in experimental models of prion diseases, in induced scrapie and in the natural disease in humans^7,15–17^. During the last decade, the role of autophagy in prion diseases has been investigated in induced murine models of prion diseases^18–21^, and new treatments for prion diseases based on the activation of the autophagic flux have been tested in cell culture^22–25^. However, the biological role of autophagy in the natural disease, or even the relationship of this process with prion-related pathology, are still poorly understood. Investigating this process in natural models such as ovine scrapie could possibly help in understanding the role of autophagy in human prion diseases, as studies on human brain samples are very few and generally suffer from small number of replicates^20^.

Autophagy requires activity of numerous proteins involved in distinct steps of this degradative route^26^. Among these, ATGs (Autophagy-Related proteins), MAP1-LC3s (Microtubule-Associated Protein 1 Light Chain 3 proteins, hereafter referred to as LC3) and p62/SQSTM1 (sequestosome 1) have been previously analysed in detail in different neurodegenerative diseases. The aim of this study was to analyse the regulation of autophagy in the CNS of sheep naturally infected with scrapie in a clinical phase. Through the combination of gene expression and immunohistochemical analysis of autophagy markers, the relationship between autophagy induction and prion pathology is presented, showing clear regional differences in autophagy regulation in response to the disease.

## Results

### Scrapie-associated neuropathology corresponds to the classical form of the disease

Prior to the analysis of autophagy, the clinical diagnosis of selected sheep was confirmed and the distribution of prion related histopathological lesions was determined. Neuropil spongiosis, intraneuronal vacuolation and PrP^Sc^ deposition were evaluated semi-quantitatively in eight brain regions: frontal cortex (Fc), basal ganglia (Bg), basal ganglia cortex (Bgc), thalamic cortex (Tc), thalamus (T), pons (P), cerebellum (Cbl) and medulla oblongata (Mo). In addition, five neuronal nuclei of the Mo were also studied: the hypoglossal motor nucleus (HMN), the dorsal nucleus of the vagus nerve (NVN), the lateral cuneate nucleus (LCN), the nucleus of the trigeminal nerve spinal tract (NTN) and the olivary nucleus (ON).

The pattern of the lesions was analysed in six controls and six clinical, scrapie-infected sheep (Supplementary Figs S1 and S2). Despite the fact that high variability was observed for all lesions within the scrapie group, statistically significant differences (P < 0.05) were detected between scrapie animals and the control group in most cases. The evaluation of haematoxylin-eosin-stained sections revealed a significant and strong increase of spongiosis and neuronal vacuolation in the T, P and Mo of the infected animals compared with controls. PrP^Sc^ immunolabelling confirmed the diagnosis of scrapie in sheep that presented neurological symptoms. Intraneuronal and neuropil PrP^Sc^ deposition was detected only in the affected animals. PrP^Sc^ immunolabelling was significantly strong in P, T and Mo, moderate in Cbl and Fc, and weak in Bg, Bgc and Tc. In Mo, the NVN displayed the highest scores for PrP^Sc^ deposition and for the other studied lesions. As expected, scrapie-related lesions positively correlated with each other (Supplementary Table S1). The observed lesion pattern corresponds to the one described for the classical form of scrapie^27^.

### mRNA expression of two autophagy markers, *ATG5* and *ATG9*, is downregulated in scrapie brains

**Figure 1:**
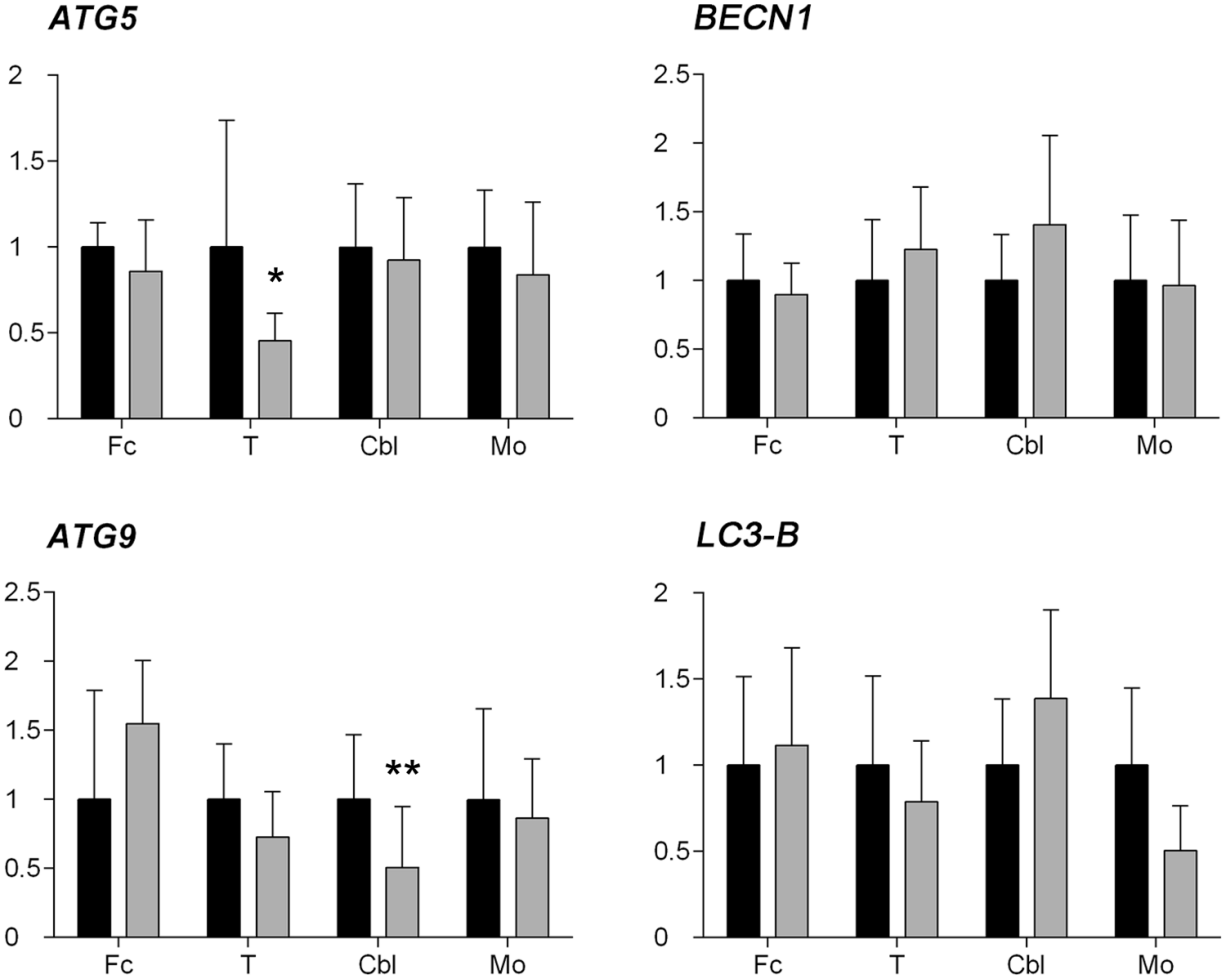
mRNA expression profiles of the *ATG5, BECN1, ATG9* and *LC3-B* autophagy-related genes. Relative expression levels in control (black bars) and scrapie-infected sheep (grey bars) in the frontal cortex (Fc), thalamus (T), cerebellum (Cbl) and medulla oblongata (Mo) are expressed as mean ± standard deviation. Results were normalized using the geometric mean of the expression of three housekeeping genes (*GAPDH*, *G6PDH* and *RPL32*). The expression values were log transformed to analyse the differences between the two experimental groups using the Student’s *t*-test (*P < 0.05 and **P < 0.01).

To investigate the regulation of the autophagy process in scrapie animals, expression of four genes (*ATG5*, *BECN1*, *ATG9* and *LC3-B*) involved in distinct steps of autophagy were quantified in Fc, T, Cbl and Mo of scrapie-infected sheep and healthy controls by quantitative real-time PCR (qRT-PCR). As shown in Fig. 1, gene expression profiles were different in each analysed area and generally few alterations were found. However, a significant downregulation was observed for *ATG5* (P < 0.05) in T and *ATG9* (P < 0.01) in Cbl of scrapie-infected animals.

### Expression of *ATG5* and *BECN1* is negatively correlated with prion deposition in thalamus of scrapie animals

To distinguish any relationship between the degree of lesion and the level of transcripts for autophagy markers, non-parametric Spearman’s rank correlation coefficients (rho, ρ) were calculated between expression levels and the scores for PrP^Sc^ deposition, neuronal vacuolation and neuropil spongiosis in the four analysed areas. Significant correlations found were mostly related with gene expression changes observed between scrapie and control animals (Supplementary Table S2) and significance was lost when scrapie sheep only were analysed. However, the strong negative correlation observed between *ATG5* and prion deposition in thalamus in the total set of individuals (ρ = −0.739, P < 0.01) was maintained in scrapie tissues (ρ = −0.833, P < 0.05). *BECN1* expression levels also correlated negatively (ρ = −0.833, P < 0.05) with PrP^Sc^ deposition in this tissue in scrapie sheep.

### Accumulation of ATG5 protein in medulla oblongata of scrapie sheep

**Figure 2:**
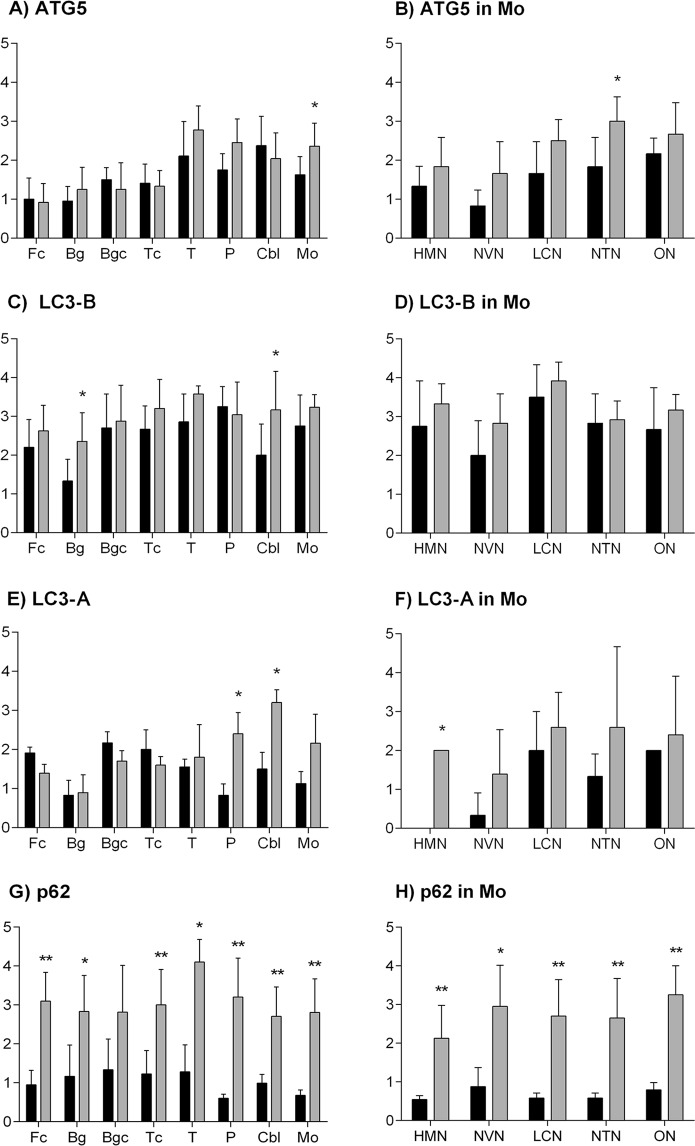
Semi-quantitative scoring of immunohistology for autophagy-related proteins. Score values of ATG5 (**A**,**B**), LC3-B (**C**,**D**), LC3-A (**E**,**F**) and p62 (**G**,**H**) (from 0: negative, to 5: staining present at its maximum intensity) evaluated in frontal cortex (Fc), basal ganglia (Bg), basal ganglia cortex (Bgc), thalamic cortex (Tc), thalamus (T), pons (P), cerebellum (Cbl) and five neuronal nuclei of the medulla oblongata (Mo): the hypoglossal motor nucleus (HMN), the dorsal nucleus of the vagus nerve (NVN), the lateral cuneate nucleus (LCN), the nucleus of the trigeminal nerve spinal tract (NTN), and the olivary nucleus (ON). Black bars: control sheep, grey bars: scrapie-infected sheep. The differences between the two experimental groups were determined using the Mann Whitney U test (*P < 0.05 and **P < 0.01).

**Figure 3:**
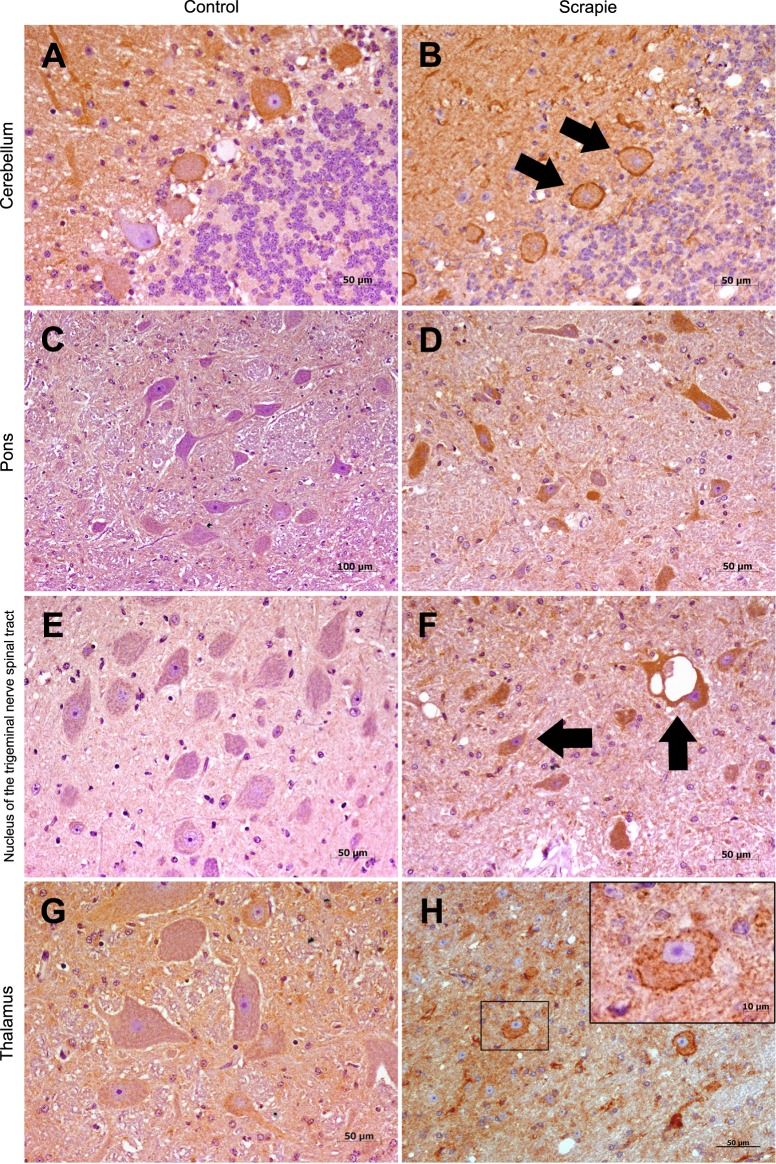
Immunostaining patterns of ATG5 protein in different CNS regions. (**A**) Strong immunostaining in cerebellum from control animals (50 µm). (**B**) Similar immunostaining in cerebellum from scrapie sheep, note the perineuronal arrangement around the Purkinje cells (arrows). No differences were observed between groups (50 µm). (**C**) Lack of intraneuronal staining in pons from control sheep (100 µm). (**D**) Intracytoplasmic immunolabelling of several neurons in pons of scrapie animals. Due to a high variability observed within these animals, this tissue did not show significant differences (50 µm). (**E**) Absence of immunostaining in the nucleus of the trigeminal nerve spinal tract (NTN) from control group (50 µm). (**F**) Diffuse punctiform immunostaining throughout the neuropil and intracytoplasmic immunolabelling of vacuolized and non-vacuolized neurons (arrows) in the NTN in the infected sheep (50 µm). In the thalamus, moderate or weak staining was observed from both control (**G**) (50 µm) and scrapie sheep (**H**) (50 µm) with no significant differences between groups. Detail of a neuron (**H**) (10 µm): ATG5 was occasionally localized in granules within the cytoplasm of some neurons, which displayed a perineuronal arrangement.

To evaluate if the transcript-level alterations were also observed in ATG5 protein levels, distribution of this marker was determined by immunohistochemistry (IHC) in eight areas of the CNS (Fc, Bg, Bgc, Tc, T, P, Cbl and Mo) in scrapie and control sheep. A distinctive band of ~32 kDa confirmed the specificity of the antibody used in Western blot analysis (Supplementary Fig. S3). In contrast to the transcript levels, ATG5 protein was significantly increased in the Mo of scrapie infected animals (P = 0.029), and specifically in the NTN (P = 0.028) (Fig. 2A,B). Accordingly, ATG5 mRNA and protein expression displayed a significant negative correlation (Spearman ρ = −0.420, P = 0.003). ATG5 immunostaining presented an intracytoplasmic and a punctiform pattern in the neuropil in several regions, such as the T, Cbl, P and Mo (Fig. 3). In these areas, ATG5 protein was occasionally stained as a diffuse spot throughout the neuropil, and in granules within the cytoplasm of some neurons. These granules usually displayed a perineuronal arrangement.

### LC3-B is overexpressed in scrapie cerebella and basal ganglia

**Figure 4:**
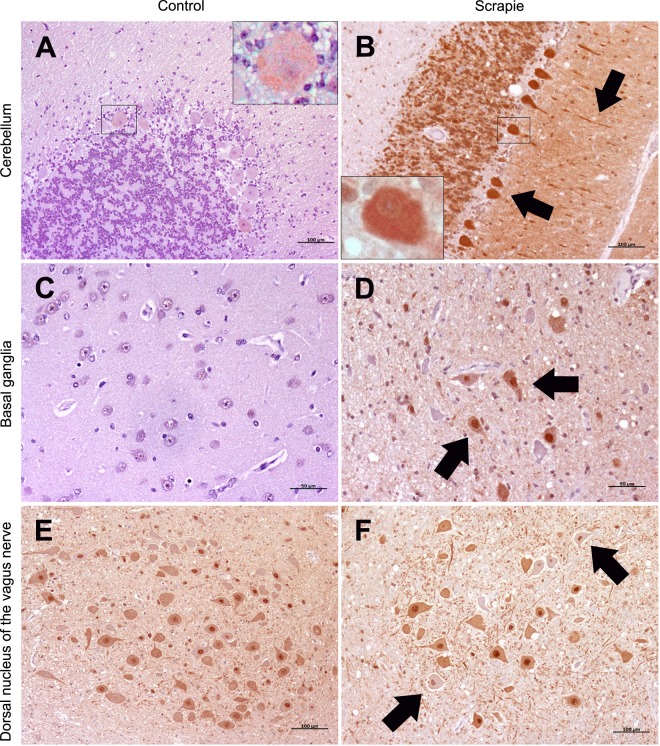
Immunostaining patterns of LC3-B protein in different CNS regions. (**A**) Weak immunostaining in the cerebellum of a non-infected control animal (100 µm). (**B**) Intense immunostaining in the cerebellum of a scrapie-infected sheep. In the cerebellar cortex, the Purkinje cells were strongly stained and their neurites were observed with intense immunolabelling (arrows) (100 µm). (**A**,**B**) Detail of LC3-B stained Purkinje cells. (**C**) Absent immunostaining in the basal ganglia from control group (50 µm). (**D**) Moderate immunostaining in the basal ganglia from scrapie animals. Unstained neurons were also observed. Note the nucleus was strongly stained (arrows) (50 µm). (**E**) Strong staining in the Dorsal nucleus of the vagus nerve (NVN) from control group (100 µm). (**F**) Similar immunostaining in the NVN from scrapie-infected sheep. The nucleus was also positive in neurons with unstained cytoplasm (arrows) (100 µm).

IHC allows the determination of small changes that could go unnoticed in gene expression analysis where mRNAs come from the different cells present in a tissue. Then, although gene expression of *LC3-B* was not modified in scrapie tissues, we next analysed the protein distribution of LC3-B by IHC. Specificity of the antibody used was tested by Western blot that revealed a single ~15 kDa band (Supplementary Fig. S3). The semi-quantitative analysis of LC3-B immunostaining showed statistically significant increase in Bg (P = 0.02) and in Cbl (P = 0.04) of scrapie-infected animals (Fig. 2C,D). This increment was confirmed comparing Cbl lysates by immunoblot. Scrapie cerebella displayed a trend (P = 0.09) to LC3-B upregulation (Supplementary Fig. S4). We did not detect LC3-II bands in the immunoblot, probably due to the dilution of signal of autophagy cells in the whole tissue. There were no significant alterations in the other tissues under study. As opposed to ATG5, LC3-B levels were not modified in the neuronal nuclei of the Mo of scrapie-infected animals and LC3-B protein levels did not correlate with those of its transcript (data not shown). Generally, LC3-B immunostaining consisted of a mild diffuse and punctiform neuropil staining (Fig. 4). In addition, neuronal cytoplasm and nuclei were generally stained positively. Even neurons with unstained cytoplasm often displayed nuclear positive immunolabelling.

### LC3-A is upregulated in specific neuronal populations of scrapie CNS

**Figure 5:**
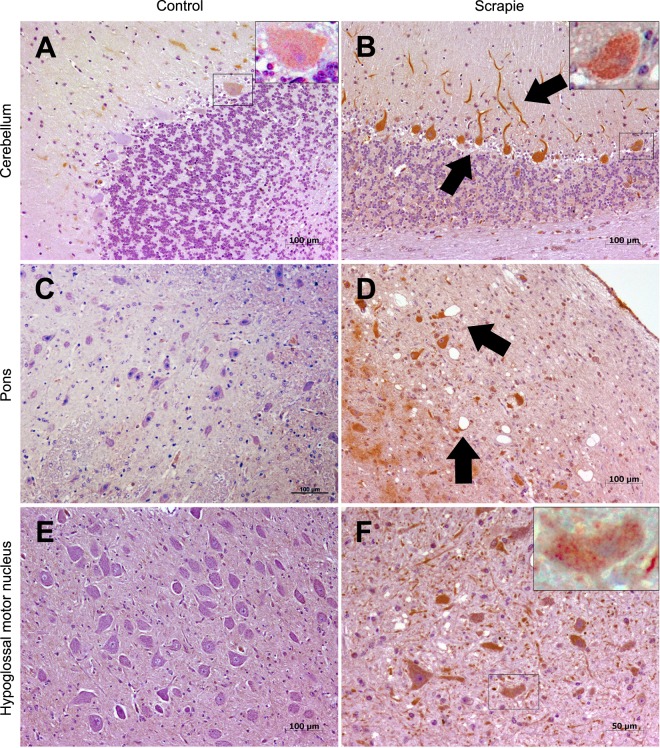
Immunostaining patterns of LC3-A protein in different CNS regions. (**A**) Moderate or weak immunostaining in the cerebellum from control animals (100 µm). (**B**) Intense immunostaining in the cerebellum from scrapie-infected sheep. In the molecular layer of the cerebellar cortex, a strong signal was observed in the Purkinje cells and their neurites (arrows) (100 µm). (**C**) Absent immunostaining in the pons from control group (100 µm). (**D**) In the pons of scrapie animals, some specific neural populations were strongly immunolabelled. Spotted and punctiform pattern was observed in the neuropil. Note the cytoplasmic immunostaining in vacuolized and non-vacuolized neurons (arrows) (100 µm). (**E**) Absent immunostaining in the hypoglossal motor nucleus (HMN) from control group (100 µm). (**F**) Diffuse punctiform immunostaining throughout the neuropil and intracytoplasmic immunolabelling of several neurons in the HMN in infected sheep (50 µm). (**A**,**B** and **F**) Detail of LC3-A stained neurons.

Although LC3-A was not analysed at the transcript level, we studied the distribution of this protein to analyse if different LC3s proteins react similarly in response to scrapie. As with the other markers, the specificity of antibody was confirmed by Western blot, displaying a band of ~15/18 kDa (Supplementary Fig. S3). In this case, LC3-A protein staining was more intense in P (P = 0.03), Cbl (P = 0.032) and HMN (expressed only in scrapie animals and not in controls) of scrapie animals (Fig. 2E,F). These differences were mainly due to an increase in the immunostaining of specific neural populations. Whereas LC3-A staining was mainly observed in the neuropil in control animals, intense neuronal immunolabelling was detected in P, Cbl and the HMN of scrapie sheep (Fig. 5). In these areas, both neurons and glial cells showing a strong intracytoplasmic immunolabelling and a diffuse punctiform immunostaining throughout the neuropil was observed. There were no significant differences in the remaining tissues between control and scrapie-infected animals. The increment of this marker in cerebella was also validated by Western blot, showing a significant increase (P < 0.001) of LC3-A signal in scrapie tissues (Supplementary Fig. S4). Similar to LC3-B, LC3-II band was not observed in the immunoblot.

### Generalized p62 accumulation in scrapie brains

**Figure 6:**
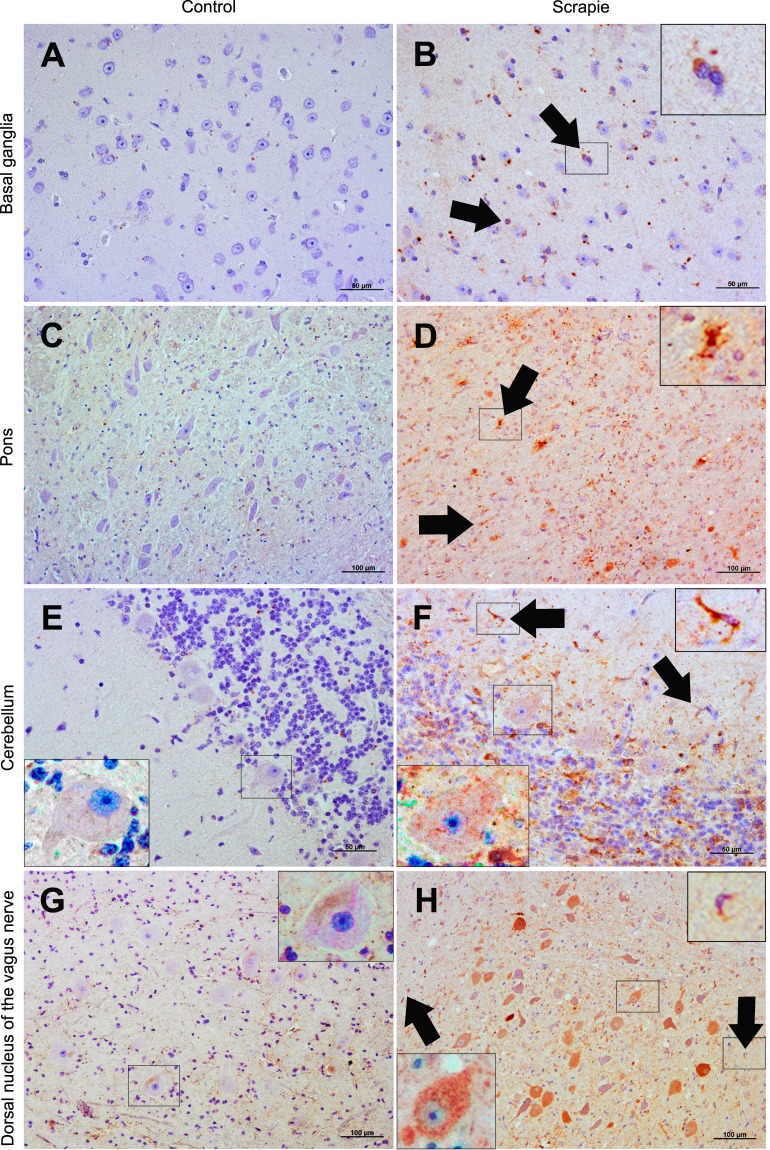
Immunostaining patterns of p62 protein in different CNS regions. (**A**) Weak granular pattern in the neuropil of basal ganglia of a non-infected control animal (50 µm). (**B**) Strong and coarse granular immunostaining in the neuropil and within the cytoplasm of cells in basal ganglia of a scrapie-infected sheep (50 µm). (**C**) Predominantly mild neuropil punctiform pattern in pons from control group sheep brain (100 µm). (**D**) Intense granular immunolabelling of the neuropil and intracytoplasmic staining of both neurons and glial cells in pons in scrapie-infected sheep (100 µm). (**E**) Weak punctiform staining in cerebellum in healthy sheep (50 µm). (**F**) Strong granular pattern and uniform intracytoplasmic staining of cells in cerebellum in scrapie-infected sheep (50 µm). (**G**) Moderate punctiform immunostaining of the neuropil in the dorsal nucleus of the vagus nerve (NVN) from control group (100 µm). (**H**) Intense staining of the neuropil and cells in the NVN in scrapie animals (100 µm). (**B**,**D**,**F** and **H**) An intense immunolabelling of cells whose morphology is compatible with glial cells (arrows and detail) was observed in scrapie-infected sheep. (**E**,**F**,**G** and **H**) Detail of p62 stained neurons.

Accumulation of p62 is commonly used to monitor autophagy impairment^28^. To investigate a possible arrest of autophagy in scrapie, we determined p62 by IHC. First, Western blot detected an expected band of ~62 kDa (Supplementary Fig. S3). Immunolabelling for p62 was remarkably intense in scrapie-infected animals, whereas little staining was detected in the control group. Scrapie sheep displayed significantly higher scores for p62 protein in all CNS areas analysed, although in Bgc this did not reach significance (Fig. 2G). Moreover, all Mo nuclei showed significant increases of this protein in the scrapie group (Fig. 2H). Staining for p62 presented a strong and coarse granular pattern within the cytoplasm of cells, and occasionally in the neuropil, in Fc, Bg, Bgc and Tc (Fig. 6). In the remaining tissues, p62 was characterised by a moderate punctiform pattern in the neuropil in healthy sheep, and by an intense granular immunolabelling of the neuropil and a uniform intracytoplasmic staining of both neurons and glial cells in scrapie animals.

### Association between ATG5 and LC3 proteins and histological features of scrapie

Protein scores for ATG5, LC3-A and LC3-B displayed positive Spearman correlations (Table 1), confirming the association detected between *ATG5* and *LC3-B* gene expression data (see above). However, in contrast to the RNA expression, LC3 proteins positively correlated with prion deposition and neuropil spongiosis both in the total set of animals and in the scrapie group (Table 1), suggesting that these proteins accumulate as the lesions related to the disease become more evident.

**Table 1:** Spearman correlation values between histological features (spongiosis, intraneuronal vacuolation and PrP^Sc^, ATG5, LC3-A, LC3-B and p62 immunostaining) in the total set of animals and in scrapie infected animals.

Autophagy markers	Spongiosis	Intraneuronal vacuolation	PrP^Sc^	ATG5	LC3-A	LC3-B	
ATG5	0.271**	0.446***	0.287**	—	0.375**	0.422***	All animals
LC3-A	0.397*	0.315*	0.373**	0.375**	—	0.330**	
LC3-B	0.332***	N.S.	0.389***	0.422***	0.330**	—	
p62	0.756***	0.478***	0.780***	N.S.	N.S.	0.440***	
ATG5	0.651***	0.571***	0.577***	—	0.487**	0.365*	Scrapie sheep
LC3-A	0.397*	N.S.	0.409**	0.487**	—	0.381*	
LC3-B	0.429**	N.S.	0.332*	0.365*	0.381*	—	
p62	0.344*	N.S.	N.S.	N.S.	N.S.	0.632***	

Correlations were estimated using the full set of data obtained in all tissues. No statistically significant correlation values are shown as N.S. (*P < 0.05, **P < 0.01; ***P < 0.001).

### p62 distribution is not related with prion deposition but with spongiform degeneration

As shown previously, p62 was overexpressed in almost all analysed areas. Besides that, p62 and LC3-B immunostaining correlated in the total set of animals (Spearman ρ = 0.440, P < 0.001) and the association was even stronger in scrapie sheep (Spearman ρ = 0.632, P < 0.001) (Table 1). As the intensity of immunostaining was not the same in the different tissues, this correlation suggests that the areas where p62 accumulates in a higher degree are the same as those where LC3-B is also strongly concentrated. Finally, p62 did not correlate with PrP^Sc^ deposition or neuronal vacuolation but displayed a relatively strong correlation with neuropil spongiosis in all animals (Spearman ρ = 0.756, P < 0.001) and weaker correlation within scrapie animals (Spearman ρ = 0.344, P < 0.05).

### Autophagy markers in cerebellar Purkinje cells

Three out of the four autophagy markers analysed by IHC displayed a significantly higher scores in scrapie cerebella and this increment seemed to be mainly due to the differences observed in immunostaining of Purkinje cells. To verify these observations quantitatively, we determined the number of stained and unstained cells in 5 independent fields for each animal. For LC3-B (Fig. 4) and LC3-A (Fig. 5), the Purkinje cells were strongly stained, and their neurites were occasionally observed with intense cytoplasmic immunolabelling. Although the percentage of stained Purkinje cells for LC3-B appeared to be higher in scrapie animals (82.0% ± 13.1) compared to controls (65.1 ± 30.5), these differences were not statistically significant. However, LC3-A was statistically increased in scrapie sheep where 94.7% ± 14.6% of the cells were positive for LC3-A compared with 68.8 ± 31.2% in controls (P < 0.01). Finally, Purkinje cells showed a moderate intraneuronal punctiform staining for p62 (Fig. 6) and the number of stained cells was greatly increased (P < 0.001) in scrapie animals (88.5 ± 20.3%) compared with controls (21.2 ± 14.5%). The graph of these results is shown in Supplementary Fig. S4.

## Discussion

Prion diseases are a group of neurodegenerative disorders characterized by accumulation of a proteinase resistant form of the prion protein (PrP^Sc^) in the CNS, which leads to spongiform degeneration and ultimate neuronal death. This process may involve several cellular pathways, including apoptosis and autophagy^7^. In previous studies assessing the molecular mechanisms of apoptosis in the CNS of sheep naturally infected with scrapie^8,9^, the induction of apoptotic pathways in scrapie brains was described^29^. However, there was no convincing evidence of a massive neuronal apoptosis, suggesting a possible activation of neuroprotective mechanisms. The aim of the present work was to analyse alterations in one of these mechanisms, autophagy, in scrapie-related neurodegeneration.

Basal levels of autophagy are important to maintain normal cellular homeostasis. However, unregulated degradation of the cytoplasmic components is likely to be deleterious. Although the CNS presents only low levels of autophagosomes under normal conditions, autophagy plays a critical role in the constitutive turnover of cytosolic contents and the removal of damaged proteins in specific neurons of the brain^30^. Defects in autophagy could impair the quality control of proteins, leading to the accumulation of toxic aggregates and subsequently, to neurodegeneration. In accordance, the presence of abnormal autophagic activity is frequently observed in neurodegenerative diseases such as Alzheimer’s disease, Parkinson’s disease, amyotrophic lateral sclerosis^30,31^. Despite the increase in autophagosomes detected in degenerating neurons^30,31^, it is still not clear how the autophagic machinery is involved during the pathogenic course of these diseases.

Autophagic vacuoles associated with prion diseases have been described in experimental models such as mice and hamsters infected with prions^15,16,21^. These vacuoles and multi-vesicular bodies also appear in prion-infected cultured neuronal cells^32^ and in brain biopsy materials of prion-infected patients^7,17^. Combining two different methodologies, qRT-PCR and IHC, we have investigated the dynamics of autophagy in scrapie-induced neuropathology through its relationship with scrapie lesions.

We carried out a first gene expression analysis of *ATG5*, *BECN1*, *ATG9* and *LC3-B* in naturally scrapie-infected sheep brains. ATG5 and ATG9 participate in the elongation^33^ and closing^34^ of the pre-autophagosomal membrane, respectively, although neither of these proteins will be present in mature autophagosomes. BECN1, on the other hand, plays a central role in coordinating the cytoprotective function of autophagy, and regulates apoptosis and other cellular processes^35^. Four different genes encode LC3s proteins^36^, of which LC3-B protein has been most widely used for tracking autophagy. LC3 proteins are expressed in most cell types as the cytosolic protein form LC3-I that, upon induction of autophagy, is conjugated to phosphatidylethanolamine to form LC3-II, a lipidated form associated with autophagic membranes^37^. Because LC3-II is a structural component of mature autophagosomes, this protein is commonly used as a specific marker for autophagy^38^. Finally, p62/SQSTM1 is a cargo receptor that acts as a substrate during autophagic degradation, which causes this protein to be degraded by autophagosomes^39^. Therefore, the evaluation of p62 accumulation may be used to estimate autophagic impairment^28^.

Downregulation of autophagy genes *beclin-1* and *Atg5* was previously described in whole brain tissues of prion-infected wild-type mice^40^. Here, transcript expression levels of genes *ATG5* and *ATG9* decreased significantly in thalamus and cerebellum, respectively, of scrapie-infected sheep, and *BECN1* expression displayed a negative correlation with prion deposition in scrapie thalamus. Since autophagy is involved in the turnover of proteins through lysosomal degradation, this process seems to be protective in various neurodegenerative processes, including prion diseases^30,41^. In this context, the downregulation of autophagy genes upon prion infection would suggest autophagy decrease, which would contribute to the accumulation of PrP^Sc^ aggregates and its consequent toxicity.

Immunohistochemical tools can be used to identify groups of cells or specific CNS regions were autophagy is altered. Contrary to the downregulation of ATG5 protein described in brains from murine models of sporadic CJD (sCJD)^18^, ATG5 expression scores in natural scrapie correlated positively with the amount of PrP^Sc^ deposits. Moreover, in contrast to its reduced transcript levels, ATG5 protein increased in one of the most affected areas of the CNS of scrapie-infected animals, the nucleus of the trigeminal nerve spinal tract of medulla oblongata. Occasionally, this protein also displayed a perineuronal arrangement within the neuronal cytoplasm. Although the subcellular localization of ATG5 is still not clear, it binds membranes and is essential for autophagy and cytoplasm-to-vacuole transport^42^. Therefore, the observed perineuronal localization of ATG5 may indicate the induction of autophagosome formation in these neurons. However, the retrograde transport of autophagosomes and their maturation to lysosomes are impaired in other neurodegenerative diseases such as Alzheimer’s disease, which results in a massive accumulation of autophagic vacuoles within degenerating neurites^43^. In this case, the combination of increased autophagy induction and defective clearance results in Aβ accumulation^44^. It is plausible that increased ATG5 immunostaining observed here in highly affected areas in scrapie brains could reflect the induction and subsequent accumulation of non-functional autophagosomes in neurites, which may contribute to the development of prion disease. This accumulation could also explain the negative correlation observed between the levels of RNA and protein expression as increased autophagic proteins could lead to downregulation of their gene expression directly or indirectly through negative feedback.

Although LC3-B has been the most studied LC3 form to monitor autophagy^38,45,46^, different subcellular distribution for LC3 proteins has been previously described in human cancer cell lines and it was suggested that autophagosomes are formed by only one of the LC3 proteins^47^. In accordance, these proteins differ somewhat in their distribution and immunohistochemical patterns in scrapie brains and it was LC3-A which showed most significant increases. Besides in the neuronal cytoplasm, LC3-B immunostaining was also positive in the nucleus, probably because of its abundant nuclear presence^48^. LC3 becomes selectively activated in the nucleus during starvation to be subsequently redistributed to cytoplasm, where it plays a central role in autophagy^49^. Although IHC does not allow the discrimination between LC3-I and LC3-II forms, a punctate pattern similar to the one observed in our work has been related with the presence of autophagosomes^50^.

As we have discussed above, autophagy impairment has been propose as a pathogenic mechanism in Alzheimer’s disease patients and murine models of sCJD where the expression of ATG proteins is reduced, while the level of LC3-II, the main autophagosomal marker, is increased^18,51,52^. In our study, immunostaining of LC3s proteins displayed a positive correlation with prion deposition and neuropil spongiosis. However, whereas LC3-A was significantly increased in both highly affected areas (medulla oblongata and pons) and cell populations with a lower degree of lesion (cerebellar Purkinje cells), LC3-B seemed to be slightly increased in most of the areas analysed in scrapie brains but was significantly upregulated only in two of the less affected areas, cerebellum and basal ganglia. Thus, the levels of these LC3s proteins as such do not reflect scrapie neuropathology in a simple manner but seem to respond differently, possibly depending on the disease stage.

The accumulation of p62 in cells and tissues from autophagy-deficient mice^53^ indicated that the degradation of p62 is dependent on autophagy. In prion research, some *in vitro* studies have proposed the enhancement of p62-activity as a possible therapeutic target for the induction of autophagy, because overexpression of p62 promotes degradation of PrP^Sc^ ^54^. However, the accumulation of p62 is generally used as a marker for inhibition or defects in autophagic activity^55^. Contrary to p62 downregulation observed in hamsters infected with prions^20^, p62 accumulates in scrapie brains in almost all CNS areas analysed, which would indicate an impairment of autophagic activity in the CNS during the course of the natural disease. In the same manner, the amino-terminally truncated prion protein PrP90-231 triggers autophagic process through the upregulation of both LC3-II and p62 *in vitro*, leading to progressive accumulation of autophagolysosomes with impaired resolution ability, resulting in prion accumulation and toxicity^23^.

In scrapie brains, p62 was generally increased but its immunostaining did not correlate with PrP^Sc^ deposition, as it was upregulated in areas with different degrees of lesions. The increment of p62 is normally associated to a reduction of autophagy, however, the increment of LC3 and p62 simultaneously has been also linked with an increment of autophagy^54^, making interpretation of results difficult. On one hand, upregulation of p62 and ATG5 in highly affected areas (medulla oblongata) could reflect an unsuccessful effort of the neurons for counteracting prion infection leading to autophagy impairment and the accumulation of non-functional autophagosomes. On the other hand, overexpression of LC3s and p62 proteins, but not ATG5, in minimally prion-affected areas of scrapie-infected animals (cerebellum and basal ganglia) suggests the activation of the autophagic machinery as a defence mechanism that protects against neurodegeneration and leads to prion clearance, as it has been proposed in murine models *in vivo*^54^, but it could also reflect a very early moment in autophagy impairment.

Moreover, in Purkinje cells of scrapie animals there was an overrepresentation of LC3-A and p62 positive stained cells compared to controls. In these cells, ATG5 remained unchanged and p62 staining was lower than in highly affected areas. This could indicate successful induction of autophagy in these cells. These results are in accordance with previous studies that indicate a remarkably efficient autophagosome formation in Purkinje cells of hamsters inoculated with 263K scrapie agent^20^. A previous study revealed intense immunolabelling of chaperones Hsp70 and Hsp90 in Purkinje cells of natural scrapie sheep^56^. Chaperones, like autophagy, are involved in the removing of altered proteins from the cell. Hence, both processes may be interwoven and involved in specific neuroprotective mechanisms in this cell type against the toxicity of PrP^Sc^. However, we cannot rule out that the changes observed constitute an early mechanism in the response to prion toxicity as cerebellum is one of the tissues that is affected later in classical scrapie. The analysis of atypical scrapie cases where PrP^Sc^ immunolabelling is particularly prominent in the cerebellum^57^ would contribute to clarify the role of autophagy in these cells.

To conclude, the presented gene expression and immunohistochemical study suggests a differential regulation of autophagic machinery in the different areas of the CNS in scrapie infected sheep used as a natural model of prion diseases. The downregulation of autophagy related genes and the increment of ATG5 and p62 suggest an arrest of the autophagy machinery in highly affected areas that could be a consequence of an impairment of neuronal function and would facilitate prion replication. Conversely, specific areas and cell populations like basal ganglia and cerebellar Purkinje cells could still have the autophagy machinery intact and the overexpression of LC3-A and p62 would facilitate prion elimination and cell survival. The impairment of autophagy could be a late effect of the disease and it should be considered when designing possible treatments directed to induce autophagy.

## Methods

### Animals and sample collection

In this work, 12 female sheep (Rasa Aragonesa breed), including six controls and six animals naturally infected with scrapie, were used. Scrapie infected animals were adults of age 56.30 ± 5.76 months, and displayed the ARQ/ARQ genotype for *PRNP* (prion protein) gene (homozygous for polymorphism at codons 136, 154 and 171), which is the most frequently observed genotype in scrapie animals from this breed^58^. These animals came from scrapie-infected flocks from several geographical regions and were diagnosed *in vivo* by the clinical signs associated with the disease. To confirm the diagnosis of scrapie post-mortem, we used IHC to detect PrP^Sc^ in the medulla oblongata with the L42 monoclonal antibody, as described in the “Histopathological and PrP^Sc^ detection studies” section below. Control animals (*n* = 6) of the same genotype, breed, sex, and similar age (48.81 ± 16.93 months) were selected from flocks belonging to scrapie-free regions. To reveal any additional polymorphisms in the *PRNP* gene, we sequenced the coding region from the experimental sheep following the previously described methods^59^. Supplementary Table S3 shows detailed characteristics of the experimental animals. Protective polymorphism at codon 141^60^ was observed in one control sheep. Variation in codon 143 displayed heterozygous and homozygous genotypes for the rare variant 143R in two control sheep. Finally, dimorphism at codon 176 was detected in other control sheep. Scrapie infected sheep did not display additional polymorphisms.

Animals were sacrificed by intravenous injection of sodium pentobarbital and exsanguination, with subsequent necropsy. Post-mortem examination of the animals did not reveal any additional pathologies. Sample collection followed the established safety guidelines. Immediately after extraction, the whole brain was divided into two sagittal halves. One of these halves was fixed in 10% neutral-buffered formalin for histopathological and immunohistochemical analysis and the other one was immediately frozen on dry ice and maintained at −80 °C temperature until RNA extraction for gene expression studies. Later, the brains were dissected in five sections to isolate the most relevant neuropathological tissues, i.e. Fc, Bg along with Bgc, T with Tc, P with Cbl, and Mo.

### Histopathological and PrP^Sc^ detection studies

Brain slices (2–3 mm) were paraffin-embedded and intraneuronal vacuolation and neuropil spongiosis were evaluated in 4 µm sections stained with haematoxylin-eosin (HE) in eight brain areas (Fc, Bg, Bgc, T, Tc, P, Cbl and Mo) and five neuronal nuclei of the Mo. IHC for PrP^Sc^ detection in the selected regions was performed as previously described^61^ using the mouse monoclonal antibody L42 [1:500 dilution at RT (room temperature) for 30 minutes (min); R-Biopharm, Darmstadt, Germany] as the primary antibody.

### Gene expression

To analyse the expression of *ATG5*, *BECN1*, *ATG9* and *LC3-B* genes by qRT-PCR, total RNA was isolated from Fc, T, Cbl, and Mo using the RNeasy Lipid Tissue Mini Kit (Qiagen, Crawley, UK) according to the manufacturer’s instructions. Tissue samples were homogenised using a TeSeE Precess 48 (Bio-Rad, CA, USA) and TURBO DNase (Ambion, Austin, TX, USA) was used to remove possible genomic DNA contamination. Complementary DNA (cDNA) was synthesized from 1 µg of total RNA using random hexamers with the Superscript First-Strand Synthesis System for RT-PCR (Invitrogen, Carlsbad, CA, USA).

Supplementary Table S4 shows the specific primers for the genes of interest used for qRT-PCR. In order to improve the accuracy of normalization we used the geometric mean of the expression of three housekeeping genes (*GAPDH*, *G6PDH* and *RPL32*) in the same sample as normalization factor. These housekeeping genes are the three most stably expressed reference genes in sheep brain^62^. Their use as internal references in previous expression studies concerning scrapie infection and the primers and PCR conditions used for their amplification have been previously described^62^.

PCR amplifications were performed in a 7500 Fast Real-Time PCR System (PE Applied Biosystems) using SYBR® Green (PE Applied Biosystems) assays. All qRT-PCR reactions were run in triplicate in a total reaction volume of 10 µl using cDNA equivalent to 10–20 ng total RNA as template. Universal amplification conditions were used, with an initial activation and cDNA denaturation step for 10 min at 95 °C, followed by 40 cycles of 3 seconds (s) at 95 °C and 30 s at 60 °C. To identify the presence of nonspecific PCR amplicons or high levels of primer dimers, we performed a dissociation curve protocol after each qRT-PCR reaction. The levels of gene expression were determined using the comparative Ct method. The Ct calculations were set automatically with the ABI-Prism 7500 software, version 2.0.1.

### Immunohistochemical determination of autophagy markers

The expression and distribution of ATG5, LC3s and p62 proteins were studied by IHC in formalin-fixed, paraffin-embedded CNS tissue sections from the clinical scrapie sheep and control animals. The tissues were pre-treated using heat-induced epitope retrieval with Tris EDTA buffer (pH 9.0) in a PTLink (Dako) at 96 °C for 20 min. Sections were incubated at 4 °C overnight for mouse monoclonal anti APG5 (C-1, sc-133158; Santa Cruz Biotechnology, 1:50) and rabbit polyclonal anti MAP-LC3α (R-23, sc-134226; Santa Cruz Biotechnology, 1:50), and 1 hour (h) at RT for mouse monoclonal anti MAP-LC3β (G-2, sc-271625; Santa Cruz Biotechnology, 1:200) and rabbit polyclonal anti p62 (PW9860; Enzo Life Sciences, 1:200). Omission of the primary antibody served as background control for nonspecific staining. The visualization system used was the enzyme-conjugated polymer EnVision (Dako EnVision anti-mouse for APG5 and MAP-LC3β; Dako EnVision anti-rabbit for MAP-LC3α and p62).

### Western blot determination of autophagy markers

Before IHC, the specificity of antibodies against ATG5, LC3-B, LC3-A and p62 was prior determined by Western blot (Supplementary Fig. S3). At least 0.5 g of the CNS tissues (Fc, T, Cbl and Mo) were homogenized each in 5 ml of Prionics® Check Western homogenization buffer (Prionics AG, Zurich, Switzerland) and centrifuged twice at 10000 × *g* for 10 min at 4 °C. The supernatants were analysed by Western blot. Briefly, 25 μg of total protein was subjected to 10% SDS/PAGE and transferred to PVDF membranes (GE Healthcare, UK). After blocking at 4 °C overnight, the membranes were incubated for 1 h at RT with the above described primary antibodies diluted in blocking buffer: anti APG5 (1:1000 dilution); anti MAP-LC3β (1:8000); anti MAP-LC3α (1:1000), and anti p62 (1:1000). In addition, goat polyclonal anti Actin (I-19, sc-1616; Santa Cruz Biotechnology, 1:10000) was used to normalize results. Next, the membranes were incubated for 1 h at RT with a HRP-conjugated secondary antibody diluted 1:4000 in blocking buffer (goat anti-mouse IgG-HRP for APG5 and MAP-LC3β, or goat anti-rabbit IgG-HRP for MAP-LC3α and p62; Santa Cruz Biotechnology, CA, USA). After washing, Western blots were developed using the ECL Plus Western Blotting system (GE Healthcare, UK) and visualized with VersaDoc imaging system (Bio-Rad).

In order to verify the differences observed by IHC for LC3-A and LC3-B and to visualize LC3-I and LC3-II bands, we compared lysates from scrapie (*n* = 4) and control (*n* = 4) cerebella. The quantification analysis was performed based on the blotting results using the ImageJ 1.4.3.67 image-analysis software package (Psion Image, NIH) following a simple method of analysis, performed by integrating the grey levels of pixels (volume) surrounded by a rectangular selection. This method is described on the ImageJ website^63^. Density of immunoreactive bands for autophagy markers was normalized for Actin density band and is reported as arbitrary units (a.u.). Data are expressed as means ± standard error.

### Data analysis

The tissue sections were examined with a ZEISS Axioskop 40 optical microscope. The extent of neuronal vacuolation, neuropil spongiosis, PrP^Sc^ deposition, as well as ATG5, LC3s and p62 immunolabelling was evaluated semi-quantitatively and scored on a scale ranging from 0 to 5 (0 = absence of lesions or immunolabelling, 5 = substantial lesions or immunolabelling throughout the region) as previously described^64–66^. Scoring for each parameter (either a lesion type or an immunolabelling pattern) of each studied area was blind performed by a pathologist making subjective evaluation. Scores for each specific area are plotted as the mean ± standard error of all individuals in each group. Histopathological and immunohistochemical differences between the experimental groups were evaluated using non-parametric Mann Whitney U test.

Relative gene expression values were log transformed and data were analysed with the Student’s *t*-test. Similarly, normalized protein expression values obtained by Western blot were compared with the Student’s *t*-test. Moreover, correlations between gene expression, protein immunolabelling and the different lesions were determined using the non-parametric Spearman’s rank correlation coefficient (rho, ρ). In all tests, the results were considered significant at P < 0.05. We used IBM® SPSS® statistics 22 software for all data analysis. Finally, to identify possible differences in LC3-B, LC3-A and p62 immunolabelling in Purkinje cells, stained and non-stained Purkinje cells were counted in five microscope areas (20× magnification) for each animal. Proportions were converted to arcsine values before applying the Student’s *t*-test.

### Ethics approval

The Ethics Committee for Animal Experiments of the University of Zaragoza (Permit Number: PI38/15) approved all procedures. The care and use of experimental animals were performed in strict accordance with the national law (R.D. 53/2013).

## Supplementary information

Supplementary material

## Data Availability

Row data from qPCR and IHC scoring are available from authors upon request.
