# Supplementary material for: Dysregulation of autophagy in the central nervous system of sheep naturally infected with classical scrapie
Source: Sci Rep. 2019 Feb 13;9:1911. doi: 10.1038/s41598-019-38500-2 (PMC6374525; doi:10.1038/s41598-019-38500-2)
Supplement: Supplementary file 1 — Supplementary material [file 41598_2019_38500_MOESM1_ESM.pdf]

Dysregulation of autophagy in the central nervous system of sheep naturally infected with classical scrapie

Óscar López-Pérez[1,2], Alicia Otero[2], Hicham Filali[2], David Sanz-Rubio[1], Janne M. Toivonen[1], Pilar Zaragoza[1], Juan J Badiola[2], Rosa Bolea[2,+], Inmaculada Martín-Burriel[1,2,*,+]

[1] Laboratorio de Genética Bioquímica (LAGENBIO), Universidad de Zaragoza, IA2, IIS Aragón, Zaragoza, 50013, Spain.

[2] Centro de Investigación en Encefalopatías y Enfermedades Transmisibles Emergentes, Universidad de Zaragoza, IA2, IIS Aragón, Zaragoza, 50013, Spain.

* minma@unizar.es

+ These authors contributed equally to this work

SUPPLEMENTARY MATERIAL

Supplementary Table S1: Spearman correlation values between scrapie-related histopathological lesions: PrP^{Sc} deposition, spongiosis and intraneuronal vacuolation.

Scrapie lesions	PrP^{Sc} deposition	Spongiosis
**Spongiosis**	0.830***	----
**Intraneuronal vacuolation**	0.818***	0.817***

(*** P<0.0001)

Supplementary Table S2: Spearman correlation values between gene expression levels and histological features in the four CNS analysed areas (medulla oblongata, thalamus, frontal cortex and cerebellum). Correlations were estimated using data from each analysed tissue independently and from the total set of animals and in scrapie infected sheep.

	**Total set of animals**			**Scrapie sheep**		
**Gene expression**	**Spongiosis**	**Intraneuronal vacuolation**	**PrP^{Sc}**	**Spongiosis**	**Intraneuronal vacuolation**	**PrP^{Sc}**
ATG5						
**Medulla oblongata**	N.S.	N.S.	N.S.	N.S.	N.S.	N.S.
**Thalamus**	-0.561▪	-0.693*	-0.739**	N.S.	N.S.	-0.833*
**Frontal cortex**	N.S.	N.S.	N.S.	N.S.	N.S.	N.S.
**Cerebellum**	N.S.	N.S.	N.S.	N.S.	N.S.	N.S.
BECN1						
**Medulla oblongata**	N.S.	N.S.	N.S.	N.S.	N.S.	N.S.
**Thalamus**	N.S.	N.S.	N.S.	N.S.	N.S.	-0.833*
**Frontal cortex**	N.S.	N.S.	N.S.	N.S.	N.S.	N.S.
**Cerebellum**	N.S.	N.S.	N.S.	N.S.	N.S.	N.S.
ATG9						
**Medulla oblongata**	N.S.	N.S.	N.S.	N.S.	N.S.	N.S.
**Thalamus**	N.S.	N.S.	N.S.	N.S.	N.S.	N.S.
**Frontal cortex**	N.S.	N.S.	0.607*	N.S.	N.S.	N.S.
**Cerebellum**	-0.719*	N.S.	-0.807**	N.S.	N.S.	N.S.
LC3-B						
**Medulla oblongata**	N.S.	N.S.	N.S.	N.S.	N.S.	N.S.
**Thalamus**	-0.583*	N.S.	N.S.	N.S.	N.S.	N.S.
**Frontal cortex**	N.S.	N.S.	N.S.	N.S.	N.S.	N.S.
**Cerebellum**	N.S.	N.S.	N.S.	N.S.	N.S.	N.S.

No statistically significant correlation values are shown as N.S. (▪P=0.05, *P<0.05, **P<0.01)

Supplementary Table S3: Age and *PRNP* genotype of the animals used

**Group**	**Animal ID**	**Age (months)**	***PRNP* genotype**
**Control**	S1 (-)	37.7	ARQ/ARQ
	S2 (-)	67.1	ARQ/$ARQK_{176}$
	S3 (-)	54.9	ARQ/ARQ
	S4 (-)	56.47	ARQ/$AF_{141}RQ$
	S5 (-)	56.56	$AR_{143}RQ$/$AR_{143}RQ$
	S6 (-)	20.16	ARQ/$AR_{143}RQ$
**Scrapie**	S7 (+)	59.86	ARQ/ARQ
	S8 (+)	60.50	ARQ/ARQ
	S9 (+)	63.83	ARQ/ARQ
	S10 (+)	50.43	ARQ/ARQ
	S11 (+)	52.06	ARQ/ARQ
	S12 (+)	51.13	ARQ/ARQ

Supplementary Table S4: Primer sequences for each gene analysed. Final concentrations of the primers in the real-time PCR reaction mix, size of the fragment amplified and GenBank accession are also given. **F: forward and R: reverse.**

Gene	Primer sequence	Concentration (nM)	Size (bp)	Accession number
ATG5	F: 5´ AAGCAACTCTGGATGGGTTTACA 3´	300	95	AM086994.1
	R: 5´ CCATTTTCTTCTGCAGGATATTCC 3´	300		
BECN1	F: 5´ CTGGACACGAGCTTCAAGATTCT 3´	200	75	AM051355.1
	R: 5´ GCTGGGCTGTGGCAAGTAAT 3´	200		
ATG9	F: 5´ TCACCACCGTCACACTCCT 3´	200	129	JQ035663.1
	R: 5´ TGGTCAGGCATGTAGTGGATG 3´	200		
LC3-B	F: 5´ CCGAGAGCAGCATCCTACCA 3´	300	87	AY570553.1
	R: 5´ AAACTTTGTTTTATCCAGGACAGGAA 3´	300		

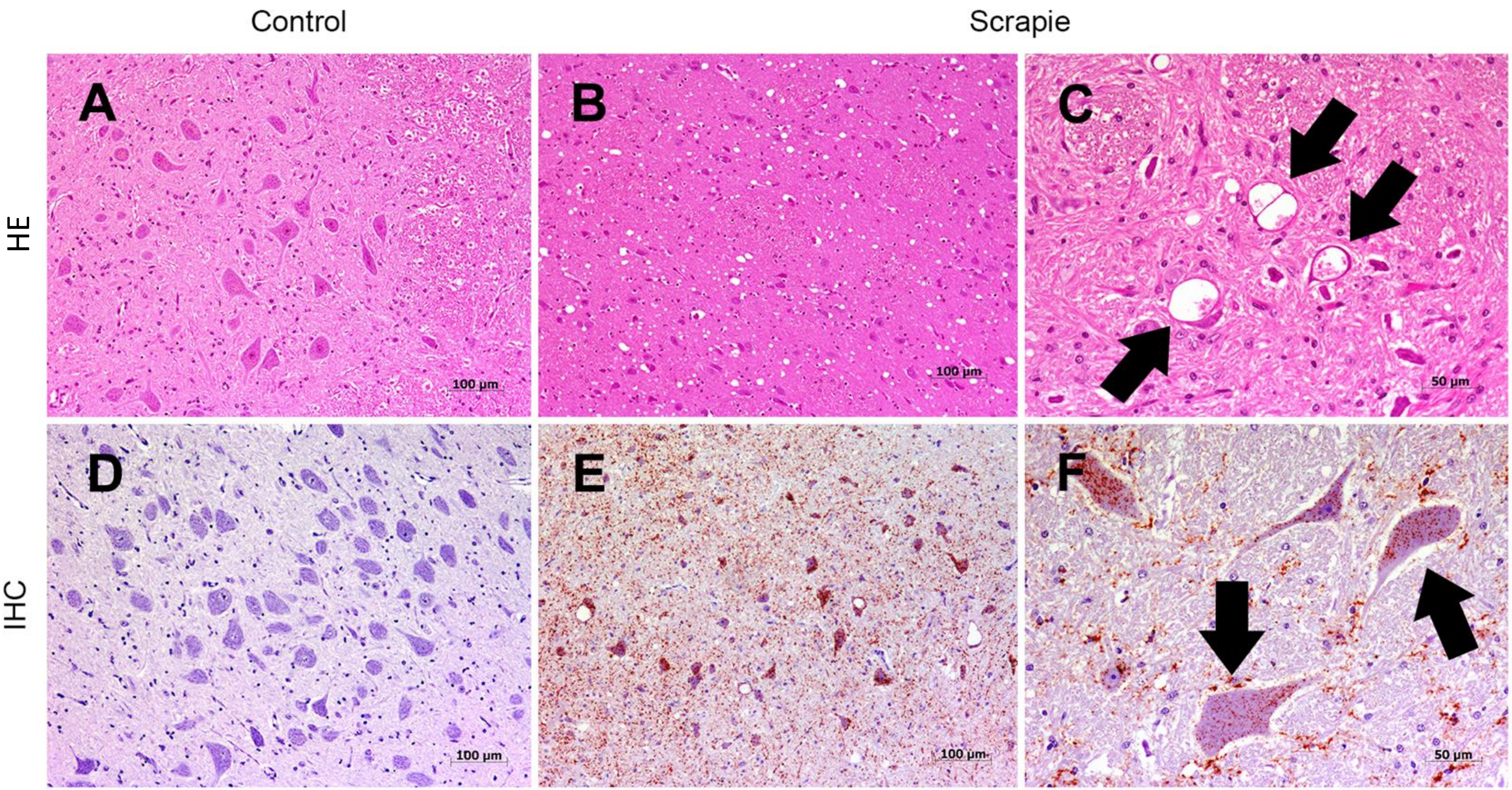

Supplementary Figure S1: Histopathological (A-C) and immunohistochemical (D-F) features from different regions of animals studied. (**A and F**) Pons. (**B**) Thalamus. (**C**) Nucleus of the trigeminal nerve spinal tract. (**D and E**) Dorsal nucleus of the vagus nerve. Detection of neuropil spongiosis and intraneuronal vacuolation was as follows: (**A**) control group showed integrity of neuropil and neuronal perikaryon (HE) (100 µm); (**B**) vacuoles in neuropil (HE) (100 µm) and (**C**) neuronal perikaryon (arrows) (HE) (50 µm) in scrapie-infected sheep. Detection of PrP^{Sc} was as follows: (**D**) controls displayed no deposition (IHC) (100 µm); infected animal tissues showed (**E**) a multigranular staining in the neuropil (IHC) (100 µm) and (**F**) neuronal perikaryon (arrows) (IHC) (50 µm).

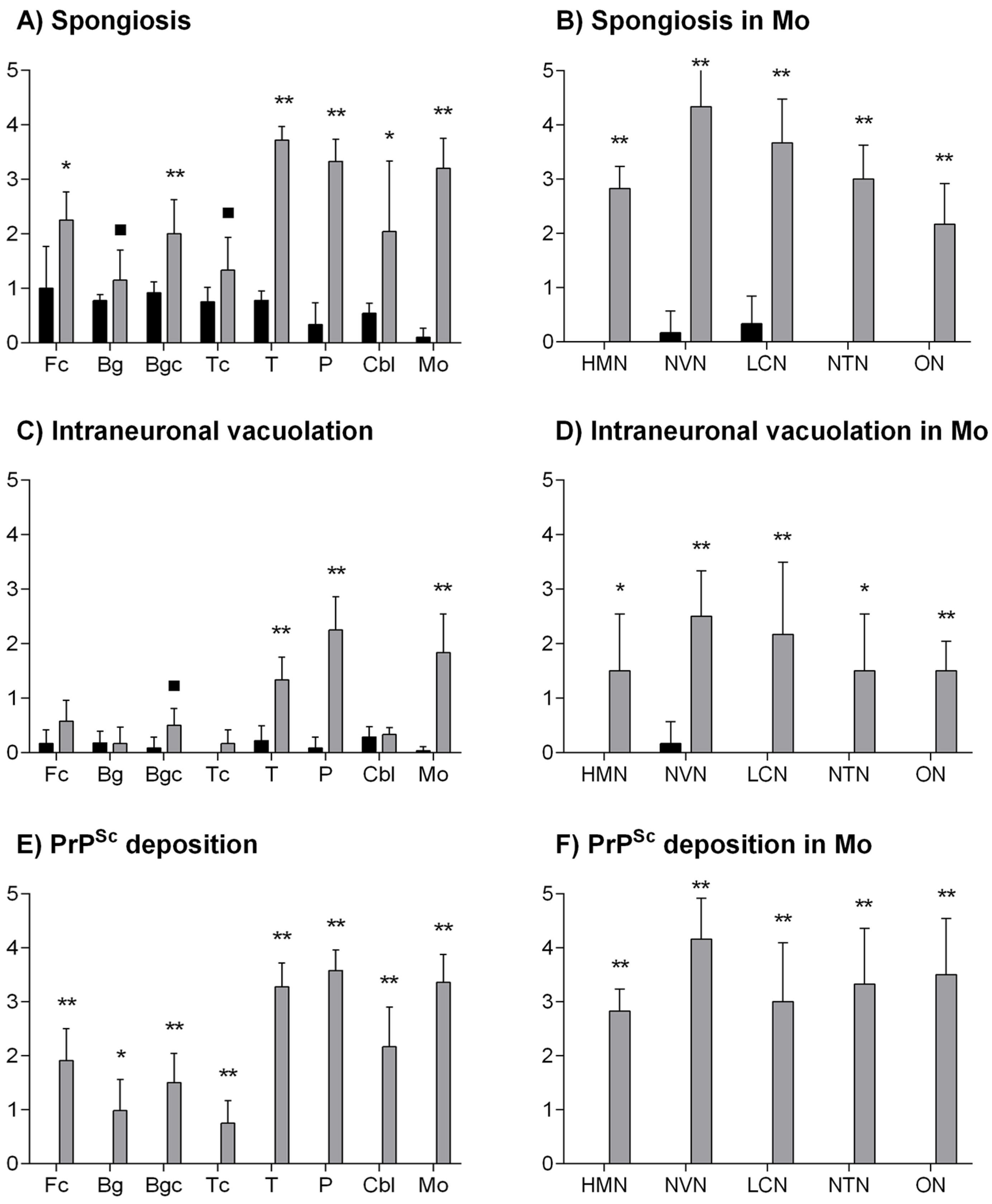

Supplementary Figure S2: Semi-quantitative assessment values of spongiosis (A-B), intraneuronal vacuolation (C-D) and PrP^{Sc} deposition (E-F). Graphics show the average score of these lesions in frontal cortex (Fc), basal ganglia (Bg), basal ganglia cortex (Bgc), thalamic cortex (Tc), thalamus (T), pons (P), cerebellum (Cbl) and five neuronal nuclei of the medulla oblongata (Mo) [the hypoglossal motor nucleus (HMN), the dorsal nucleus of the vagus nerve (NVN), the lateral cuneate nucleus (LCN), the nucleus of the trigeminal nerve spinal tract (NTN), and the olivary nucleus (ON)] of control (black bars) and scrapie-infected sheep (grey bars). Scores range from 0 (negative) to 5 (lesion/staining present at maximum intensity). Significant differences were determined using the Mann Whitney U test (■P=0.05, *P<0.05 and **P<0.01).

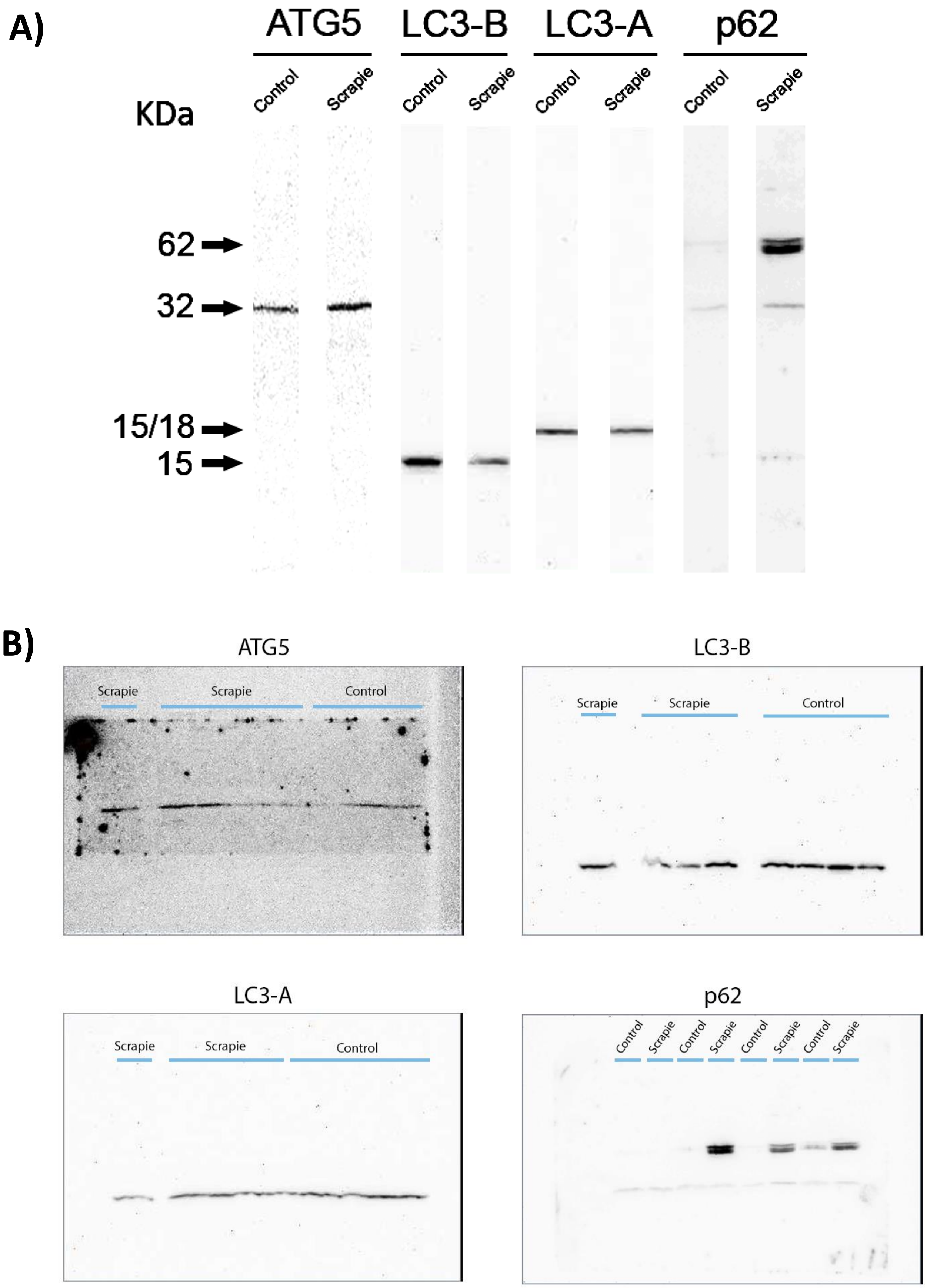

Supplementary Figure S3: Specificity of antibodies. (**A**) Membranes showing the specificity of antibodies against ATG5, LC3-B, LC3-A and p62 proteins in ovine thalamus detected by Western blot. Each protein was cropped and grouped from different parts of the same gel. Distinctive bands of ~32 kDa, ~15 kDa, ~15/18 kDa and ~62 kDa confirmed the specificity of the antibody used against ATG5, LC3-B, LC3-A and p62, respectively. (**B**) Full-length membranes with no high-contrast of ATG5, LC3-B, LC3-A and p62 proteins in thalamus in control and scrapie-infected sheep.

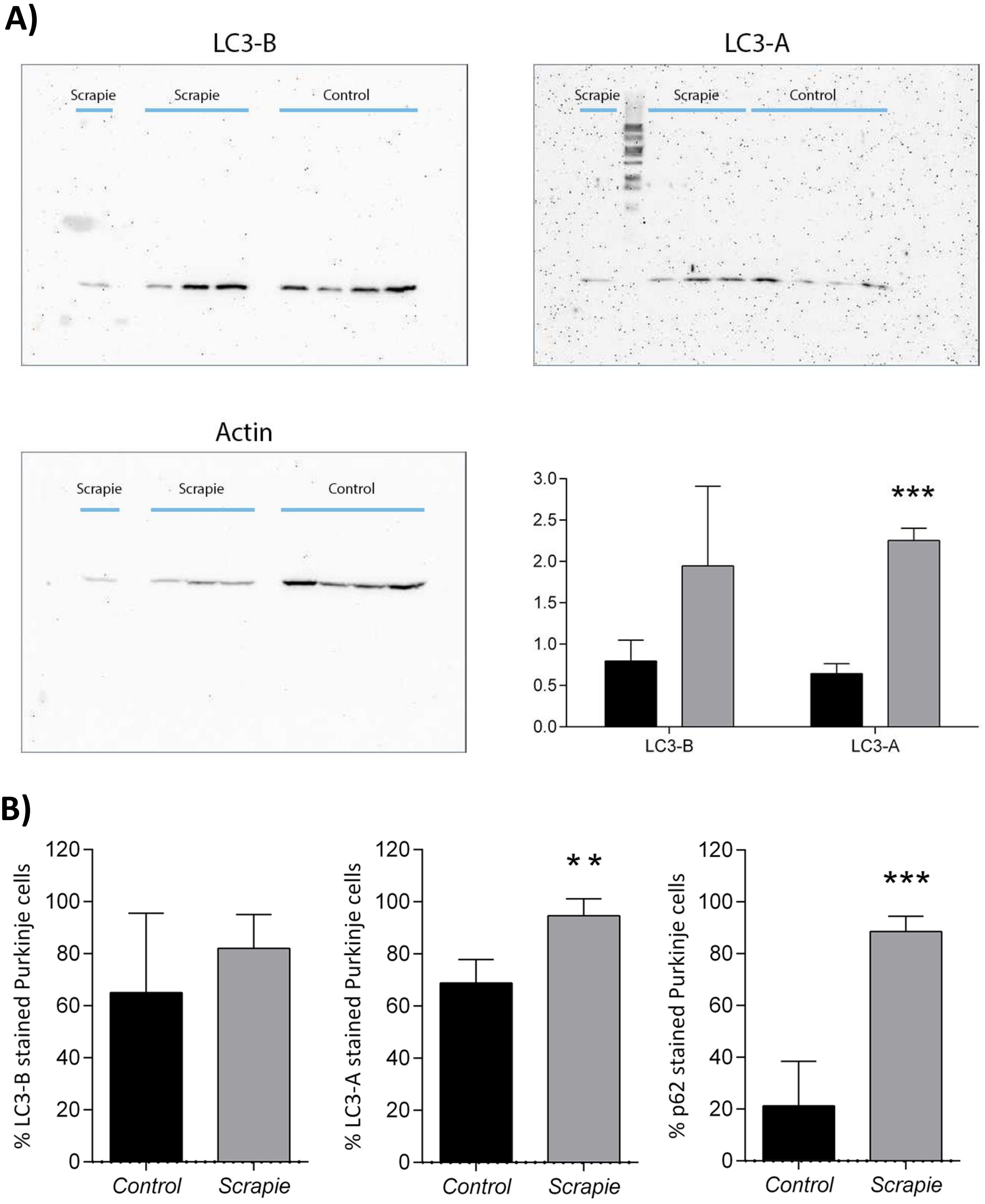

Supplementary Figure S4: (A) Western blot quantification of LC3-B and LC3-A expression in cerebellum and (B) percentage of LC3-B, LC3-A and p62 positively stained Purkinje cells. (**A**) Density of immunoreactive bands for LC3-B and LC3-A was normalized for Actin density band and is reported as arbitrary units (a.u.). Data are expressed as means ± standard error. Scrapie cerebella (grey bars) displayed a trend to LC3-B upregulation (P=0.09) and a significant increase of LC3-A (P<0.001). In any case, we did not detect LC3-II bands in the Western blot, probably due to the dilution of signal of autophagy cells in the whole tissue. (**B**) For LC3-B, no significant differences were observed between stained Purkinje cells in control and scrapie-infected sheep. The increment was statistically significant for LC3-A (P<0.01) and for p62 (P<0.001). Significant differences were determined using the Student´s *t*-test (**P<0.01 and ***P<0.001).
